# Optimization of Ultrasound-Assisted Extraction of Anthocyanins from Mulberry, Using Response Surface Methodology

**DOI:** 10.3390/ijms12053006

**Published:** 2011-05-10

**Authors:** Tang-Bin Zou, Min Wang, Ren-You Gan, Wen-Hua Ling

**Affiliations:** 1 Department of Nutrition, School of Public Health, Sun Yat-sen University, Guangzhou 510080, China; E-Mails: zoutb@163.com (T.-B.Z.); wangmin1605@tom.com (M.W.); ganry_zsu@yahoo.cn (R.-Y.G.); 2 Guangdong Provincial Key Laboratory of Food, Nutrition and Health, Guangzhou 510080, China

**Keywords:** ultrasound-assisted extraction, anthocyanins, mulberry, response surface methodology

## Abstract

Mulberry is one of the most widely used traditional Chinese medicines. Anthocyanins are the main bioactive components of mulberry, and possess important biological activities, such as antimicrobial, anti-inflammatory and antioxidant activities. This study investigated the ultrasound-assisted extraction (UAE) of anthocyanins from mulberry by using response surface methodology (RSM). The extraction conditions associated with anthocyanin yield, including extraction solvent, liquid-to-solid rate, temperature and extraction time, are discussed. The optimal conditions obtained by RSM for UAE from mulberry include 63.8% methanol contains 1% (v/v) trifluoroacetic acid (TFA), 43.2 °C temperature, 23.8 (v/w) liquid-to-solid ratio, and 40 min time for the maximum yield (64.70 ± 0.45 mg/g). The results indicated that the UAE can be an effective method for the extraction of some active components from plant materials.

## 1. Introduction

Mulberry, the fruit of *Morus alba*, is commonly used in Chinese medicines due to a variety of pharmacologic effects [1]. Anthocyanins, the flavonoid most consumed by human beings [2], are abundant in various colorful fruits, vegetables, red wine and grains [3,4]. Numeral data indicated that anthocyanins exhibit a wide range of biological activities including antimicrobial [5], anti-inflammatory [6], antioxidant [7], and antimutagenic properties [8]. Anthocyanins richly exist in mulberry, meanwhile cyanidin-3-glucoside and cyanidin-3-rutinoside have been reported to be the most abundant ones [9–12].

Extraction is a very important stage in the isolation, identification, and use of anthocyanins [13]. To describe the extraction mechanism in the literature, Fick’s second law of diffusion is usually used [14]. The recovery of anthocyanins is commonly performed through a solvent-extraction procedure and the solvent type, solvent concentration, liquid-to-solid ratio, temperature, and time are important parameters to be optimized [15]. In order to seek more efficient methods, solvent consumption should be decreased, extraction time should be shortened, and extraction yield should be increased. Various novel extraction techniques have been developed for the extraction of some active components from plants, such as ultrasound-assisted extraction (UAE), supercritical fluid extraction, enzymatic extraction, and soxhlet extraction [16–19]. Among these, UAE is an inexpensive, simple, and efficient extraction technique. The enhancement in extraction obtained by using ultrasound is mainly attributed to the effect of acoustic cavitations produced in the solvent by the passage of an ultrasound wave [20]. Ultrasound also exerts a mechanical effect, allowing greater penetration of solvent into the tissue, increasing the contact surface area between the solid and liquid phase. As a result, the solute quickly diffuses from the solid phase to the solvent [21].

Response surface methodology (RSM) is an effective statistic technique for optimizing complex processes [22]. It has been successfully demonstrated that RSM can be used to optimize the total flavonoid compound from many medicinal plants [23]. In the present study, anthocyanins were extracted by UAE and quantified by high-performance liquid chromatography with diode array detection (HPLC-DAD). UAE parameters such as methanol concentration, extraction temperature, and liquid-to-solid ratio were optimized using RSM, in order to obtain the optimal conditions for the extraction of anthocyanins from mulberry. The crude extract obtained can be used either in some mulberry-related health care products or for further isolation and purification of specific anthocyanin. Thus, the results obtained will be helpful for the full utilization of mulberry.

## 2. Results and Discussion

### 2.1. Chromatographic Results

**Figure 1.**
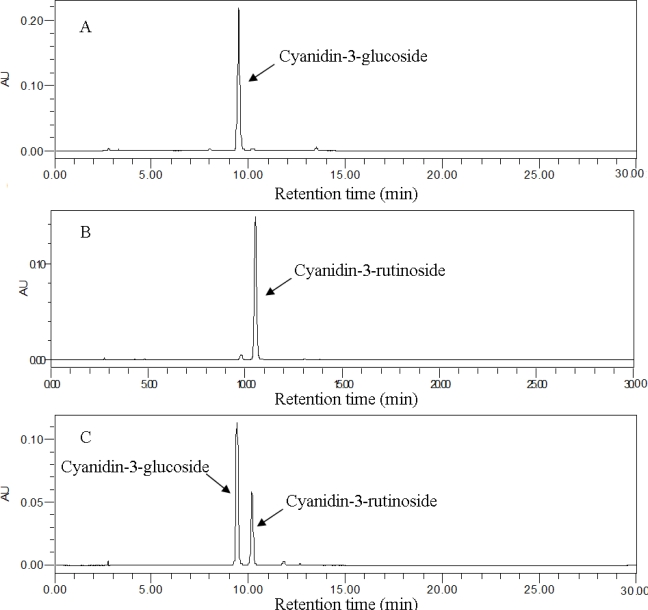
The chromatograms of cyanidin-3-glucoside (**A**), cyanidin-3-rutinoside (**B**) standard substance and mulberry extract (**C**) at λ = 520 nm.

The chromatograms of standard substance and the sample are shown in Figure 1. The authentic cyanidin-3-glucoside and cyanidin-3-rutinoside had a retention time of 9.4 min (Figure 1A) and 10.3 min (Figure 1B), respectively. The chromatogram of ultrasonically extracted sample is shown in Figure 1C. Anthocyanin yield was quantified by using calibration curves and expressed as mg/g (other anthocyanins were expressed as the amount of cyanidin-3-glucoside).

### 2.2. Selection of Solvent

**Figure 2.**
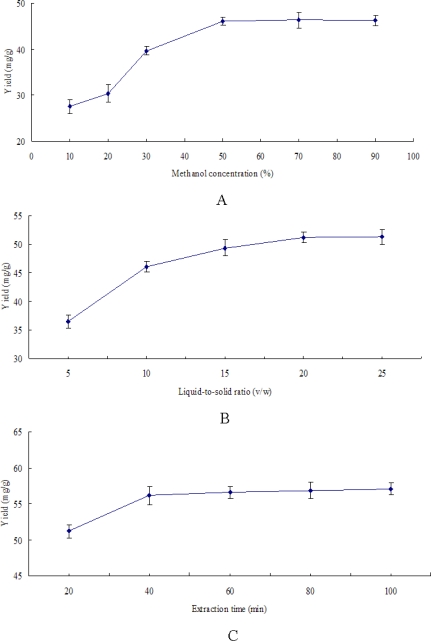

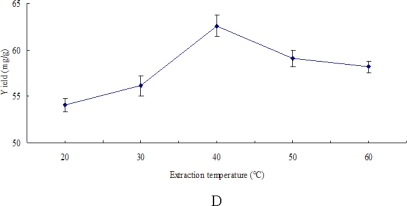
The effects of extraction parameters on anthocyanin yield: (**A**) Effect of methanol concentration on anthocyanin yield. Other conditions were fixed at 10 liquid-to-solid ratio, 30 °C extraction temperature and 20 min extraction time; (**B**) Effect of liquid-to-solid ratio on anthocyanin yield. Other conditions were fixed at 50% methanol, 30 °C extraction temperature and 20 min extraction time; (**C**) Effect of time on anthocyanin yield. Other conditions were fixed at 50% methanol, 20 liquid-to-solid ratio, 30 °C extraction temperature; (**D**) Effect of temperature on anthocyanin yield. Other conditions were fixed at 50% methanol, 20 liquid-to-solid ratio, 40 min extraction time.

The selection of extraction solvent is the first crucial step for parameters optimization. Anthocyanins are normally extracted with acidified solvents under mild conditions since they are reactive compounds and sensitive to pH changes [24]. In this study, acidified methanol (1% TFA, v/v) was employed as extraction solvent [25]. Furthermore, in order to investigate the effect of methanol concentration on the anthocyanin yield, various methanol concentrations were prepared as solvent, and the results are shown in Figure 2A. The anthocyanin yield was positively associated with the percentage of methanol when the concentration increased from 10% (27.56 ± 1.53 mg/g) to 50% (46.12 ± 0.82 mg/g). However, there was only a slight increase from 50% to 90%. Therefore, taking into account the cost and yield, 50% acidified methanol was chosen for the following experiments.

### 2.3. Volume of Solvent

Liquid-to-solid ratio is another important factor in the process of conventional extraction. Generally speaking, a larger solvent volume can dissolve constituents more effectively, leading to an enhancement of the extraction yield [26]. However, this will induce the waste of solvent. On the contrary, lower levels of solvent will result in the lower yield of the objective constituents [27]. Therefore, the choice of a proper solvent volume is significant. In this experiment, the suitable liquid-to-solid ratio was evaluated.

Figure 2B shows the effect of liquid-to-solid ratio on the extraction yield of anthocyanins. Under the fixed conditions of other factors, it could be observed that the extraction efficiency positively increased with the liquid-to-solid ratio, especially when the ratio increased from 5:1 (36.42 ± 1.15 mg/g) to 20:1 (51.20 ± 0.92 mg/g). Thus, the 20 liquid-to-solid ratio was chosen in the following experiments.

### 2.4. Extraction Time

Time duration can influence the extraction yield as well [28]. Before the establishment of equilibrium for the objective constituents in and out of plant cells, the yield of extraction increases with time. However, it cannot increase after the establishment of equilibrium [29]. In order to further research the influence of time on the extraction yield, different time points were tested from 20 to 100 min. Figure 2C shows the extraction results carried out under different time duration. The yield increased quickly with the time and reached 56.14 ± 1.20 mg/g at 40 min, since the extraction yield was almost constant from 40 to 100 min.

### 2.5. Extraction Temperature

Temperature is also an important factor in the extraction of heat sensitive compounds. Along with the increase of temperature, the solvent diffusion rate and the mass transfer intensification result in the dissolution of objective components. Meanwhile, the dissolution of impurities can also increase, and some thermal labile components such as anthocyanins can decompose [29]. In this study, extraction was carried out at different temperatures (20–60 °C) while other extraction parameters were constant. The effect of temperature on the extraction yield of anthocyanins is shown in Figure 2D. The yield significantly increased from 54.09 to 62.58 mg/g as the temperature increased from 20 to 40 °C, then began to decrease as the temperature increased from 40 to 60 °C, due to the degradation of anthocyanins. Thus, 40 °C is the preferable temperature for anthocyanin extraction.

### 2.6. Optimization of the Yield of Anthocyanins

The anthocyanin yield of mulberry was further optimized through the RSM approach. A fixed extraction time (40 min) was chosen. The coded and actual levels of the three variables in Table 1 were selected to maximize the yield. Fifteen experiments were designated, in which 12 were factorial experiments and three were zero-point tests performed to estimate the errors.

Table 2 shows the treatments with coded levels and the experimental results of anthocyanin yield of mulberry. The yield ranged from 45.63 to 63.42 mg/g. The maximum yield was recorded under the experimental conditions of *X*_1_ = 70%, *X*_2_ = 40 °C and *X*_3_ = 25. By applying multiple regression analysis on the experimental data, the response variable (yield) and the test variables are related by the following second-order polynomial equation:

$$\begin{array}{lll} Y & = & 62.17+4.08X_{1}+2.76X_{2}+2.17X_{3}-1.22X_{1}X_{2}\\ & & +1.26X_{1}X_{3}+1.27X_{2}X_{3}-3.96X_{1}^{2}-7.05X_{2}^{2}-2.28X_{3}^{2} \end{array}$$

Table 3 shows the analysis of variance (ANOVA) for the regression equation. The linear term and quadratic term were highly significant (*P* < 0.01). The lack of fit was used to verify the adequacy of the model and was not significant (*P* > 0.05), indicating that the model could adequately fit the experiment data.

The adequate precision measures the signal to noise ratio. A ratio greater than 4 is desirable. In this study, the ratio was found to be 28.99, which indicates that this model can be used to navigate the design space. The value of adjusted R-squared (0.9836) for the equation is reasonably close to 1, indicated a high degree of correlation between the observed and predicted values, therefore the model is suitable. A very low value of coefficient of the variance (C.V.%) (1.37) clearly indicated a very high degree of precision and reliability of the experimental values.

**Figure 3.**
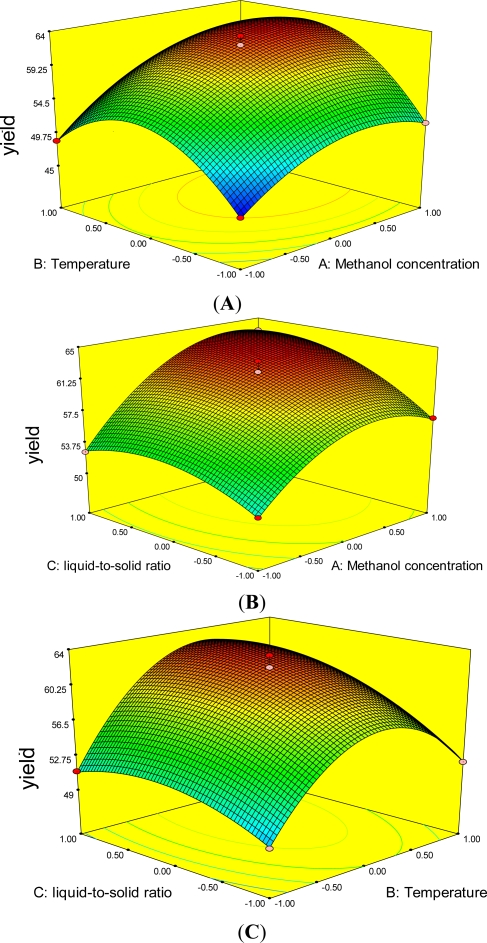
Response surface graphs for the effects of methanol concentration, temperature and liquid-to-solid ratio on anthocyanin yield of mulberry extract: (**A**) Methanol concentration (*X*_1_) and temperature (*X*_2_); (**B**) Methanol concentration (*X*_1_) and liquid-to-solid ratio (*X*_3_); (**C**) Temperature (*X*_2_) and liquid-to-solid ratio (*X*_3_).

Three-dimensional response surface plots are presented in Figure 3. An increase of methanol concentration (*X*_1_) and liquid-to-solid ratio (*X*_3_) result in an increase of anthocyanin yield to a maximum at a certain level, while an increase of temperature (*X*_2_) results in an initial increase of anthocyanin yield that then decreases when the temperature continues to rise.

The optimal values of the selected variables were obtained by solving the regression equation. After calculation by Design Expert software, the optimal conditions of anthocyanin extraction were 63.8% methanol (1% TFA, v/v), 43.2 °C extraction temperature, 23.8 liquid-to-solid ratio, and 40 min extraction time, with the corresponding *Y* = 64.84 mg/g. To confirm these results, tests were performed in triplicate under optimized conditions. The anthocyanin yield was 64.70 ± 0.45 mg/g, significantly higher than when using maceration extraction by ethanol (23.30 mg/g) [30], which clearly showed that the model fitted the experimental data and therefore optimized the anthocyanin extraction procedure from mulberry.

## 3. Experimental Section

### 3.1. Chemicals and Reagents

Cyanidin-3-glucoside and cyanidin-3-rutinoside standard substances were kindly provided by Polyphenol AS (Sandnes, Norway). Methanol, trifluoroacetic acid (TFA), and formic acid (analytical grade) were purchased from Guangzhou Chemical Industry (China). Acetonitrile (HPLC grade) was obtained from Fisher Scientific (Fairlawn, NJ, USA). The water was obtained by a purification system and filtrated through a 0.45 μm millipore filter (Pall Life Sciences, Ann Arbor, MI).

### 3.2. Plant Material

Mulberry (da-10) was obtained in March 2010 from markets in Guangzhou (Guangdong Province, China). The samples were dried in a lyophilizer (Labconco, USA), then ground and sifted for homogenization and stored at −80 °C to avoid compounds degradation [29].

### 3.3. Ultrasound-Assisted Extraction

The ultrasound-assisted extraction (UAE) was carried out in an ultrasonic device (KJ1004B, Kejin Instrument Company, China) with an ultrasound power of 200 W and frequency of 40 kHz, equipped with a digital timer and a temperature controller.

The dried powder of mulberry (about 0.5 g) was accurately weighed, and placed in a capped tube, then mixed with an appropriate amount of extraction solution. After wetting plant material, the tube with suspension was immersed into water in the ultrasonic device, and irradiated for the predetermined extraction time [31]. After ultrasonic extraction, the sample was centrifuged at 8000 rpm for 10 min, and then the supernatant was collected and diluted with eluent. All samples were filtered through a 0.45 μm syringe filter (Pall Life Sciences, Ann Arbor, MI, USA).

### 3.4. Experimental Design

The extraction parameters were optimized using response surface methodology (RSM) [22]. A Box-Behnken experiment was employed in this regard. Methanol concentration (*X*_1_), extraction temperature (*X*_2_) and liquid-to-solid ratio (*X*_3_) were chosen for independent variables. The range and center point values of the three independent variables presented in Table 1 are based on the results of preliminary single factor experiments. The experimental design consists of 12 factorial experiments and three replicates of the central point. Anthocyanin yield was selected as the responses for the combination of the independent variables given in Table 2. Experimental runs were randomized, to minimize the effects of unexpected variability in the observed responses. The variables were coded according to the following equation:

$$x=(X_{i}-X_{0})/\Delta X$$

where *x* is the coded value, *X_i_* is the corresponding actual value, *X*_0_ is the actual value in the center of the domain, and Δ*X* is the increment of *X_i_* corresponding to a variation of 1 unit of *x* . The mathematical model corresponding to the Box-Behnken design is:

$$Y=b_{0}+\sum_{i=1}^{3}b_{i}X_{i}+\sum_{i=1}^{3}b_{ii}X_{i}^{2}+\sum_{i=1}^{2}\sum_{m=i+1}^{3}b_{im}X_{i}X_{m}$$

where *Y* is the dependent variable (yield), *b_0_* is the model constant, *b_i_*, *b_ii_* and *b_im_* are the model coefficients. They represent the linear, quadratic and interaction effects of the variables. Analysis of the experimental design data and calculation of predicted responses were carried out using Design Expert software (Version 7.1.6, Stat-Ease, Inc., Minneapolis, MN, USA). Additional confirmation experiments were subsequently conducted to verify the validity of the statistical experimental design.

### 3.5. HPLC Analysis

A Waters (Milford, MA, USA) e2695 separations module with a Waters 2998 diode array detector was used. An elite® C18 column (250 mm × 4.6 mm, 5 μm) and an auto-injector were used. The analysis of anthocyanins was performed using acetonitrile as eluent A and 10% formic acid in water (10:90, v/v) as eluent B. The gradient elution program was performed as follows: 0–15 min, from 5 to 15% A; 15–21 min, from 15 to 28% A; 21–22 min, from 28 to 40% A; 22–24 min, from 40 to 60% A, and then return to the initial conditions for 3 min, followed by an isocratic elution for 3 min before the next injection. Eluates were monitored at λ = 520 nm, column temperature was 30 °C, flow rate was 1.0 mL/min, and injection volume was 10 μL.

### 3.6. Statistical Analysis

All the experiments were carried out in triplicate, and the results were expressed as means ± SD (standard deviation). Statistical analysis was conducted with SPSS 17.0 software (version 17.0, SPSS Inc., USA). A value of *P* < 0.05 was considered statistically significant.

## 4. Conclusions

An UAE method has been developed for the extraction of anthocyanins from mulberry. Ultrasonic wave is a powerful tool, which can efficiently improve the extracting performance of anthocyanins. The RSM was successfully employed to optimize the extraction and several experimental parameters have been evaluated. The results showed that methanol concentration, extraction temperature and liquid-to-solid ratio all had significant effects on the extraction rate of anthocyanins. The best combination of response function was 63.8% methanol (1% TFA, v/v), 43.2 °C temperature, 23.8 liquid-to-solid ratio, and 40 min extraction time with ultrasonic irradiation. Under the optimal conditions, the yield of anthocyanins reached 64.70 ± 0.45 mg/g powder. The results obtained are helpful for the full utilization of mulberry, which also indicated that the UAE is a powerful tool for the extraction of important phytochemicals from plant materials.

## Figures and Tables

**Table 1.** Coded and actual levels of three variables.

**Independent variables**	**Coded Levels**		
	−1	0	1
Methanol concentration (*X*_1_)	30	50	70
Temperature (*X*_2_)	30	40	50
**Liquid-to-solid ratio (*X*_3_)**	**15**	**20**	**25**

**Table 2.** Response surface design and experimental data.

**Test set**	**Coded levels**			**Anthocyanin yield (mg/g)**
	*X*_1_	*X*_2_	*X*_3_	
1	1	0	1	63.42
2	0	1	−1	52.09
3	1	−1	0	51.24
4	1	0	−1	56.73
5	−1	0	1	52.62
6	0	−1	−1	49.06
7	0	−1	1	51.05
8	−1	0	−1	50.98
9	−1	1	0	48.65
10	0	1	1	59.16
11	−1	−1	0	45.63
12	1	1	0	59.14
13	0	0	0	62.13
14	0	0	0	63.37
15	0	0	0	61.02

**Table 3.** Analysis of variance (ANOVA) for the regression equation.

**SD**	**SS**	**DF**	**MS**	***F* value**	***p* value**
Model	485.93	9	53.99	94.32	<0.0001
*X*_1_	133.25	1	133.25	232.79	<0.0001
*X*_2_	60.83	1	60.83	106.27	0.0001
*X*_3_	37.80	1	37.80	66.04	0.0005
*X*_1_*X*_2_	5.95	1	5.95	10.40	0.0233
*X*_1_*X*_3_	6.38	1	6.38	11.14	0.0206
*X*_2_*X*_3_	6.45	1	6.45	11.27	0.0202
*X*_1_^2^	57.77	1	57.77	100.92	0.0002
*X*_2_^2^	183.67	1	183.67	320.87	<0.0001
*X*_3_^2^	19.20	1	19.20	33.54	0.0022
**Lack of it**	**0.098**	**3**	**0.033**	**0.024**	**0.9937**

SD: sources of deviation; SS: sum of squares; DF: degree of freedom; MS: mean square.
